# Regulating Anger under Stress via Cognitive Reappraisal and Sadness

**DOI:** 10.3389/fpsyg.2017.01372

**Published:** 2017-08-14

**Authors:** Jun Zhan, Xiaofei Wu, Jin Fan, Jianyou Guo, Jianshe Zhou, Jun Ren, Chang Liu, Jing Luo

**Affiliations:** ^1^Beijing Key Laboratory of Learning and Cognition, Collaborative Innovation Center for Capital Education Development, Department of Psychology, Capital Normal University Beijing, China; ^2^Department of Psychology, Queens College, City University of New York, New York NY, United States; ^3^Key Laboratory of Mental Health, Institute of Psychology, Chinese Academy of Sciences Beijing, China; ^4^Beijing Advanced Innovation Center for Imaging Technology, Capital Normal University Beijing, China; ^5^Department of Psychology, Zhejiang Normal University Jinhua, China; ^6^School of Psychology, Nanjing Normal University Nanjing, China

**Keywords:** stress, anger, cognitive reappraisal, sadness induction, salivary cortisol

## Abstract

Previous studies have reported the failure of cognitive emotion regulation (CER), especially in regulating unpleasant emotions under stress. The underlying reason for this failure was the application of CER depends heavily on the executive function of the prefrontal cortex (PFC), but this function can be impaired by stress-related neuroendocrine hormones. This observation highlights the necessity of developing self-regulatory strategies that require less top-down cognitive control. Based on traditional Chinese philosophy and medicine, which examine how different types of emotions promote or counteract one another, we have developed a novel emotion regulation strategy whereby one emotion is used to alter another. For example, our previous experiment showed that sadness induction (after watching a sad film) could reduce aggressive behavior associated with anger [i.e., “sadness counteracts anger” (SCA)] (Zhan et al., 2015). Relative to the CER strategy requiring someone to think about certain cognitive reappraisals to reinterpret the meaning of an unpleasant situation, watching a film or listening to music and experiencing the emotion contained therein seemingly requires less cognitive effort and control; therefore, this SCA strategy may be an alternative strategy that compensates for the limitations of cognitive regulation strategies, especially in stressful situations. The present study was designed to directly compare the effects of the CER and SCA strategy in regulating anger and anger-related aggression in stressful and non-stressful conditions. Participants’ subjective feeling of anger, anger-related aggressive behavior, skin conductance, and salivary cortisol and alpha-amylase levels were measured. Our findings revealed that acute stress impaired one’s ability to use CR to control angry responses provoked by others, whereas stress did not influence the efficiency of the SCA strategy. Compared with sadness or neutral emotion induction, CER induction was found to reduce the level of subjective anger more, but this difference only existed in non-stressful conditions. By contrast, irrespective of stress, the levels of aggressive behavior and related skin conductance after sadness induction were both significantly lower than those after CER induction or neutral emotion induction, thus suggesting the immunity of the regulatory effect of SCA strategy to the stress factor.

## Introduction

Previous studies have suggested that cognitive reappraisal successfully down-regulates anger by reframing individuals’ interpretations of an angry situation or event. However, anger is commonly considered as a negative emotion that is difficult to regulate because it must be regulated with particular urgency in daily life (Tavris, 1984; Mauss et al., 2007). As one of the most frequently experienced negative emotions (Averill, 1983), anger is positively associated with reactive (i.e., provoked, defensive, and retaliatory) forms of aggression (especially when anger is approach-oriented; Blanchard and Blanchard, 1984; Bushman et al., 2001; Hubbard et al., 2010). Recent studies have begun to reveal the underlying reason that negative emotions such as anger or fear are so difficult to regulate. These studies suggest that stress destroys the function of cognitive regulation. In studies of mental orientation, people who were more sensitive to environmental demands (i.e., more easily placed in a state of stress) and who lacked self-control resources were more likely to experience anger (Hortensius et al., 2012). Anger regulation deficits have been widely observed in patients with post-traumatic stress disorder (PTSD) with high levels of anger (Chemtob et al., 1997; Heesink et al., 2015). Thus far, however, studies that have examined the direct relationship between stress and cognitive emotion regulation (ER) have been limited. Raio et al. (2013) suggested that the successful implementation of cognitive ER relies on the advanced function of the prefrontal cortex (PFC), which might be damaged by the deleterious effects of stress. Catecholamines (e.g., norepinephrine) and cortisol-release activated by stress might impair the cognitive functions of the PFC, thereby undermining cognitive regulation (Raio et al., 2013). Therefore, ER strategies that are less reliant on the PFC might be more suitable than normal down-regulating strategies (e.g., cognitive reappraisals) with regard to changing negative responses to emotional arousal under stress.

**FIGURE 1:**
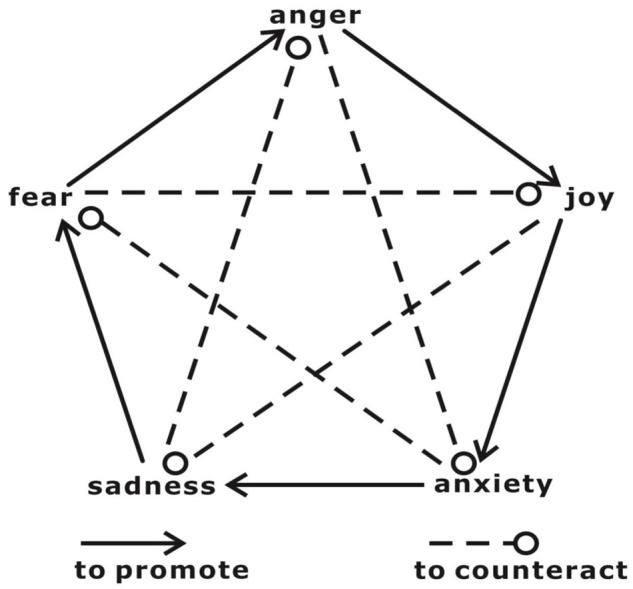
The relationships among mutual promotion and mutual restraint and the emotions of joy, thinking/anxiety, sadness, fear, and anger.

In contrast to cognitive regulation, which emphasizes the role of cognition in executing top-down ER, the “sadness counteracts anger” SCA strategy, a novel strategy based on the theories of traditional Chinese philosophy and medicine, provides an alternative approach that might overcome the deficits in the cognitive ER in stressful situations. Theories of traditional Chinese philosophy and medicine view different types of mental states and emotions as having mutual promotion and counteraction (allelopathy) relationships (**Figure 1**) (Zhan et al., 2015). One example is the hypothesis of SCA. Classic traditional Chinese medicine has recorded cases of patients who had illnesses caused by anger and who were cured by inducing sadness (Jiang and Wei, 1996). These cases demonstrate that sadness might be able to dispel the consequences of anger. The mechanism through which SCA can be understood based on the different neural mechanisms associated with sadness and anger. A recent meta-analysis of brain responses to specific emotions found that anger preferentially engaged cortical processes that support an “external orientation/object-focused” schema characterized by goal-driven responses in which objects and events in the world are in the foreground. In contrast, sadness engages cortical patterns that support an internal orientation/homeostatic-focused schema characterized by an orientation toward immediate somatic or visceral experience that prioritizes the processing of interceptive and homeostatic events (Thayer et al., 2012; Roy et al., 2014; Wager et al., 2015). The strategy based on the concept that SCA primarily refers to passively listening sad music or viewing sad movie to evoke sadness, which likely requires less cognitive control than approaches that require participants to intentionally recall a given autobiographic event or generate a cognitive reappraisal. In addition, the SCA strategy does not mean that sadness is better than anger or that anger should be replaced with sadness. Rather, this strategy implies two major points: First, it proposes that sadness is a potentially efficient emotion to counteract previously evoked anger and aggressive behavior; second, if sadness can be evoked in a harmless and effortless way (e.g., by watching sad movies), then it might be an alternative approach (relative to cognitive reappraisal) to regulate anger. Our recent study provided behavioral evidence supporting the hypothesis that SCA and found that subsequently induced sadness reduced an angry individual’s aggressive behavior (but not angry feelings) more than neutral or fear emotions (Zhan et al., 2015). In that study, participants were first provoked by reading negative feedback on their viewpoints or by watching anger-inducing movie clips. Then, they were assigned to three groups, and they viewed video clips to induce sadness, fear, or neutral emotions. We found that participants produced less aggressive behaviors when sadness was subsequently induced (i.e., SCA) but a higher level of anger when fear was induced (i.e., “fear promotes anger”). The regulatory effects of sadness counteracting anger in that study might be related to the distraction effect in which individuals attempt to shift their attention from one topic or task to another, which reduces negative emotions (McRae et al., 2010). However, this distraction perspective cannot account for group differences across the three conditions because the distraction that the three groups experienced was approximately comparable in its duration and task features (i.e., passively viewing short movie clips). The means of interaction between the emotion to be regulated (i.e., anger) and the subsequently induced emotion (i.e., fear or sadness) produced outcomes in which sadness counteracted anger and fear promoted anger. Furthermore, we speculated that sadness did not decrease self-reported anger because of the distraction feature of the SCA strategy (in particular, that sadness subsequently decreased existing anger-related responses). In contrast to a top-down regulatory strategy (e.g., cognitive reappraisal) that entails the explicit alteration of the self-relevant meaning (an appraisal) of an emotion-inducing stimulus (Ochsner et al., 2002; Etkin et al., 2015), the direct target of sadness induction is not the participant’s conscious attitude toward a previous anger-inducing event. In other words, although the participants’ induced anger was not relieved, their aggressive behavior decreased. However, this effect does not mean that sadness is only effective for reducing aggressive behaviors and not for alleviating subjective feelings. Besides, we are now considering to apply this SCA strategy to control the anger and aggression of the automobile drivers in Beijing (there are about 30–40% of the drivers reported the experience of “road rage” because of the traffic jams and the frequently encountered impolite driving manners) by having people to listen to sad music during driving. In fact, the sad music could be even more beneficial to make the drivers to focus on their driving and result in less accident than other types of music for example the happy ones (Pêcher et al., 2009). A discussion of this issue is presented in the “Discussion” Section.

The present research sought to directly contrast the effectiveness of cognitive reappraisal and sadness induction to relieve anger under non-stress and stress situations. The major assumptions of the present study were two-fold: (1) In the non-stress condition, both cognitive reappraisal and sadness induction after an anger-evoking situation can significantly decrease anger-related responses; and (2) in a stress situation, cognitive reappraisal fails to reduce anger, whereas sadness might still counteract anger, as in the non-stress condition.

In the present study, self-reported anger, aggressive behavior and skin conductance (SC) were assessed as indices of anger and emotional arousal (Lobbestael et al., 2008). Although different from anger, aggressive behavior is often positively correlated with subjective angry feelings (Bushman et al., 1999; Parrott and Giancola, 2007) and often presented as a consequence of anger (Bandura et al., 1963; Novaco, 1994; Lieberman et al., 1999). SC variation is predictive of emotional arousal and positively associated with aggression (Bradley and Lang, 2000; Lorber, 2004; Murray-Close et al., 2012). In response to a stressor, two major biological stress systems are activated: the sympathetic nervous system (SNS) during the period immediately following the onset of the stressor and, with a time delay, the hypothalamic-pituitary-adrenal (HPA) axis (Sapolsky et al., 2000; Arnsten, 2009; Ulrich-Lai and Herman, 2009). To ensure that our stress manipulation elicited HPA axis activity, we collected salivary samples and analyzed them for cortisol concentrations, a hallmark of HPA axis activity (Sapolsky et al., 2000; Arnsten, 2009; Arnsten et al., 2012). In fact, cortisol release is preceded and triggered by the earlier release of catecholamines, which is driven by SNS arousal and released rapidly after a stressor to accelerate the preparatory responses to stress. To measure this response, we assayed salivary α-amylase (sAA), which serves as a marker of noradrenergic activity (van Stegeren et al., 2006; Thoma et al., 2012).

## Materials and Methods

### Participants

A total of 204 undergraduate and graduate students from universities in Beijing participated in this study. Participants were ineligible for the study if they were pregnant, menstruating, ill or becoming ill, or taking any antidepressant or antianxiety medications. All participants signed an informed consent document approved by Institutional Review Board at Capital Normal University’s Committee on Activities Involving Human Subjects and were compensated ¥30 for their participation. The data from 24 participants were excluded from the final analysis because the saliva samples of 13 failed to be centrifuged, and 11 correctly guessed the purpose of the experiment and were not induced for sadness or cognitive reappraisal by watching corresponding videos. Our final analysis included 180 healthy participants (66% women) with a mean age of 20.76 years (*SD* = 1.73; range: 19∼24 years).

### Experimental Design and Procedures

#### Overview of the Experimental Procedure

**FIGURE 2:**
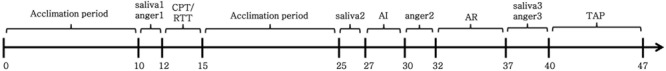
Schematic of the experimental procedure and the timeline of the neuroendocrine assessments. CPT, cold pressor task; RTT, room temperature task; AI, anger induction; AR, anger regulation (sadness induction/cognitive reappraisal); TAP, Taylor aggression paradigm. Saliva1, saliva2 and saliva3, represent the first (at baseline), second (after stress/non-stress induction) and third (after anger regulation) saliva collection times, respectively. Anger1, anger2 and anger3 represent the first (at baseline), second (after anger induction) and third (after anger regulation) anger assessment times, respectively.

To test our predictions, the present study used a 2 (stress state: stress condition and non-stress condition) × 3 (regulation strategy: sadness, cognitive reappraisal, and neutral mood induction) between-group design. The entire experimental procedure consisted of three stages (**Figure 2**). First, the participants were randomly assigned to the stress or non-stress conditions. Then, anger was induced in all of the non-stressed and stressed participants. Third, sadness, cognitive reappraisal, or neutral mood inductions were conducted separately among the provoked participants for both the stress and non-stress conditions. During the experiment, self-reported stress levels were collected two times (before and after the stress/non-stress induction) to examine the stress induction manipulation. Three evaluations of self-reported anger were administered at baseline, after anger induction and after the cognitive reappraisal/sadness mood induction/neutral mood induction (the procedures for inducing the sadness/neutral emotion or cognitive reappraisal are hereinafter referred to as the anger regulation phase) to examine the manipulation of anger induction and investigate the effectiveness of anger regulation after the assignment of cognitive reappraisal or sadness or neutral mood induction. Moreover, we collected the participants’ saliva at baseline, after stress/non-stress induction and after anger regulation to measure the cortisol and sAA concentrations, which are reliable biological stress markers of HPA axis and SNS activity, respectively. Moreover, SC was recorded throughout the experiment and served as an index of anger arousal. Finally, aggressive behavior was assessed using the Taylor Aggression Paradigm (TAP). A full oral debriefing (with a probe for suspicion) followed. The experimenter also asked the participants to maintain the secrecy of the experimental procedures to prevent future participants from acquiring knowledge regarding this experiment.

##### Stress manipulation

The cold pressor test (CPT) was used to induce individual stress in the present study. This test is among the most commonly used laboratory stressors (Lovallo, 1975; Lupien et al., 2007; Lee et al., 2013; Raio et al., 2013). Participants in the stress condition were required to submerge their right hand to their elbow in a 0∼2°C ice-water bath for 3 min, whereas the participants in the non-stress condition immersed their right arms in room temperature water for 3 min. The CPT reliably elicits two major stress systems of the body, the SNS and the HPA axis (Sapolsky et al., 2000; Arnsten, 2009; Ulrich-Lai and Herman, 2009).

##### Anger induction

We modified the anger-induction procedure with reference to the mutual-evaluation paradigm developed by Bushman et al. (1999, 2001), Bushman (2002), which has been widely used in studies on anger and aggressive behavior. Before the day of the formal experiment, each participant wrote a paragraph focusing on a popular topic in Chinese society (e.g., the new marriage law on property division in divorce) and to exchange his or her views on the subject with another participant (who did not exist) by e-mail. Furthermore, the participants were asked to evaluate the view of the other participant using a score ranging from -10 (very poor) to 10 (very good) and to provide a brief comment, which was sent to the experimenter’s e-mail. During the anger induction phase in the formal experiment, the experimenter showed the participant an extremely negative evaluation of his or her viewpoint. The participant was told that this evaluation was made by the participant who exchanged views with him or her but was actually prepared in advance by the experimenter. The data of participants who expressed suspicion about our anger induction procedure were not included in the experiment for further analysis.

##### Cognitive reappraisal, sadness emotion induction, and neutral mood induction assignment

Three videos were used to induce individuals’ sadness, cognitive reappraisal and neutral moods. The sad movie clip (duration: 2 min 16 s; from the movie “Mom Love Me Once Again”; intensity, *M* = 3.17, *SD* = 1.56) and the neutral movie clip (duration: 2 min 17 s; from the movie “Computer Repair”; intensity, *M* = 1.0625, *SD* = 0.25) were extracted from the Chinese Emotional Visual Stimulus (CEVS) database (Xu et al., 2010). In contrast to cognitive reappraisals, which are commonly administered with the experimenter’s instruction, a cognitive reappraisal video (duration: 3 min 6 s) was created to match the experimental manipulations of sadness and neutral emotion induction in the presentation of videos and content (i.e., the visual, sound and language stimulation in the three videos involved experiences in daily life). This cognitive reappraisal video was recorded in a simulated psychological counseling room with a Canon video tape recorder. In the video, a “psychological counselor” (played by a psychology Ph.D. student) introduced the cognitive reappraisal strategy based on rational-emotive therapy (RET). Importantly, because the anger induction procedure was based on a false insult to the participants, the experimenter pretended to be naïve to the truth and the participants’ anger throughout the experiment. Thus, the content of the cognitive reappraisal video did not directly contrast with the prior negative event in the anger induction phase; rather, it included several digestible examples (including anger events) in daily life that aimed to induce the participants to reframe the prior anger event in the anger induction procedure and to change their current anger response. To avoid arousing the participants’ suspicion with regard to the prior anger induction procedure, the participants were informed that the cognitive reappraisal video taught material that needed to be assessed before being put online. To further ensure the effectiveness of the cognitive reappraisal video before the formal experiment, we asked a random sample of 34 undergraduate students (non-psychology students) to watch this video; 94% of these participants considered the video content as straightforward and conducive to regulating negative mood. While watching all of the videos, the participants were asked to be as attentive to the videos as possible, to express their natural feelings and not suppress any emotion. After completing all of the experimental procedures and inquiries, the participants were immediately asked whether the content of the cognitive reappraisal video was understandable and useful for helping them to regulate their feelings of anger evoked by the negative comments of their partner. If the participants did not understand or accept the content of the cognitive reappraisal video or sadness was not induced, then these participants were excluded from the formal data analysis.

##### Self-reported stress

To assess the effectiveness of the stress induction, all participants reported how stressed they were before and immediately after the CPT/control task on a reverse-scored 5-point Likert scale ranging from 1 (extreme stress) to 5 (no stress), where 1: very tense; 2: tense; 3: between tense and relaxed; 4: relaxed; and 5: very relaxed.

##### Self-reported anger

The subjective feeling of anger was measured using the hostility subscale of the revised Multiple Affect Adjective Checklist (MAACL; Zuckerman and Lubin, 1985; Bushman et al., 2001). In the Chinese version of the MAACL (Zhang, 1991), the hostility subscale contains 22 adjectives, including 11 words that are positively associated with anger (*irritable, cruel, jealous, disgruntled, indignant, impatient, hostile, irritated, violent, furious*, and *exasperated*) and 11 words that are negatively associated with anger (*gracious, easy-going, good-natured, helpful, friendly, courteous, gentle, pleasantly agreeable, kind, affable*, and *cooperative*). All of the participants assessed these 22 adjectives according to their current feeling and selected each positive anger word or deselected each negative anger word. Each word accumulated one point; thus, the final scores were the sum of the total points for the selected positive anger words and for the unselected negative anger words. A high total score indicated a high level of anger. The self-reported anger evaluations were administered three times: (a) at baseline (i.e., at the beginning of the experimental session), (b) after the anger-induction procedure, and (c) after the cognitive reappraisal, sadness mood induction, or neutral mood induction assignment.

##### Aggressive behavior measure

The measurement of aggression was presented as a competitive reaction time task based on the paradigm developed by Taylor (1967) that has been widely used in studies of anger and aggressive behavior (Taylor, 1967; Bushman and Baumeister, 1998; Muller et al., 2012; Elson et al., 2014; Watkins et al., 2015). In this task, participants were first led to believe that they were playing against a participant who had previously given them insulting feedback (refer to the anger induction procedure). This competitive reaction time task required that participants press a button as quickly as possible for each trial. The participant who was slower received a blast of white noise (similar to radio static) through headphones. Before each trial, the participants designated the noise intensity that their partner would receive, if they won the competition. This intensity ranged from 60 (Level 1) to 105 decibels (Level 10, approximately the same volume as a smoke alarm). A non-aggressive no-noise setting (level 0) was also offered. The participants were also permitted to control how long their partner would hear the noise, from 0.5 s (Level 1) to 5 s (Level 10). The two measures were standardized and summed to form a single measure of interpersonal aggression (Bushman and Baumeister, 1998). The “partner” set random noise levels throughout the task. A 105-decibel noise is uncomfortable but not painful or harmful.

##### Salivary cortisol and sAA determinations

To examine sAA and salivary cortisol stress responses, saliva samples were collected using cotton swabs (Sarstedt Ltd., Germany). Participants were instructed to gently chew on the swab for 1∼2 min. We ensured that baseline cortisol and sAA levels were stable by collecting the first salivary sample 10 min after the participants arrived at the laboratory. Salivary samples were taken three times during the course of the experiment: at baseline (i.e., 10 min after the participant’s arrival), 10 min after stress or control manipulation (when cortisol was expected to rise in the stress condition), and after anger regulation (approximately 20 min after stress or control manipulation). All samples were immediately stored in a sterile tube and kept at -20°C until analysis. Salivary cortisol and sAA were assayed using ELISA kits (Cortisol Parameter Assay Kit, R&D Systems, Inc., United States and Canada; Human Amylase ELISA Kit, Assaypro LLC., St. Charles, MO, United States).

##### SC assessment

To measure the physiological activation of anger arousal during the experimental procedure, SC was sampled at a rate of 1000 Hz and recorded using an MP150 system (BIOPAC Systems, Inc., Goleta, CA, United States). For SC recordings, two shielded Ag-AgCl electrodes filled with standard NaCl electrolyte gel were placed on the palmar sites of the middle phalanges of the second and third fingers of the left hand. Data were collected with AcqKnowledge software (Biopac Systems) and inspected visually during the entire experiment. Offline, the signal was amplified 10× and passed through a rolling 1-Hz low pass filter to remove movement artifact. Mean SC data are reported in microsiemens (μS) with a refresh rate of 1 s. Measurements were taken continuously during the 5-min period of the baseline stage and the 5-min period including the anger regulation stage and the subsequent self-reported emotion evaluation stage. The mean SC values of these two periods were used in the final analysis.

## Results

### Subjective Feeling of Stress

The 2 (time: at baseline and after stress/non-stress induction) × 2 (stress state: stress induction or non-stress induction) × 3 (regulation strategy: sadness induction, reappraisal induction, or neutral mood induction) repeated-measures ANOVA on self-reported stress showed a significant main effect of time [*F*_(1,174)_ = 25.411, *p* < 0.001, *η^2^* = 0.127] but non-significant main effect of regulation strategy [*F*_(2,174)_ < 1, *p* > 0.05, η^2^ = 0.010]. The interaction among time, stress state and regulation strategy as well as the interaction between time and regulation strategy were not significant [*F*_(2,174)_ < 1, *p* > 0.05, η^2^ = 0.005; *F*_(2,174)_ < 1, *p* > 0.05, η^2^ = 0.002, respectively], whereas the interaction between time and stress state was significant [*F*_(1,174)_ = 48.960, *p* < 0.001, η^2^ = 0.220].

**FIGURE 3:**
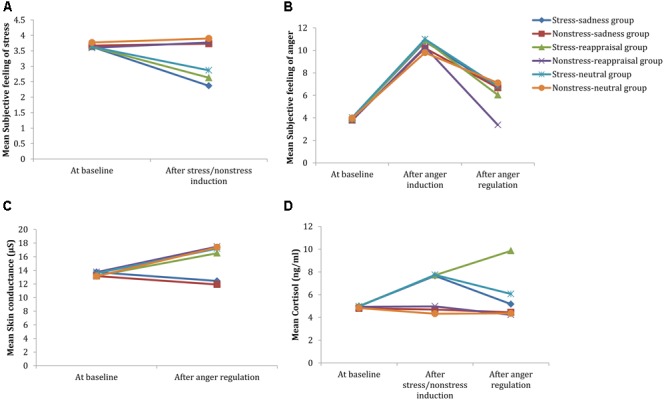
Comparisons of emotional, SC and cortisol changes among the six conditions. Participants’ subjective feelings of stress before stress induction and after stress/non-stress induction are shown in **(A)**. Participants’ subjective feelings of anger at baseline, after anger induction and after anger regulation are shown in **(B)**. Participants’ SC at baseline and after anger regulation are shown in **(C)**. Participants’ salivary cortisol levels at baseline, after stress/non-stress induction and after anger regulation are shown in **(D)**.

Regarding the significant interaction time and stress state, a simple effect analysis (adjusted using the Šidák correction) focusing on the time differences showed that the self-reported stress scores after stress induction were significantly lower than those at baseline (*p* < 0.001); however, no significant differences were observed between the self-reported stress scores after non-stress induction and those at baseline (*p* > 0.05; see **Figure 3A** and **Table 1**). A simple effect analysis (adjusted using the Šidák correction) focusing on the stress state differences did not show significant differences at baseline (*p* > 0.05), but the self-reported stress scores after stress induction were significantly lower than those after non-stress induction (*p* < 0.001; see **Figure 3A** and **Table 1**). Because the stress scale in our study was reverse scored, these results indicate that the stress manipulation was successful at increasing individuals’ subjective feelings of stress.

**Table 1:** Subjective feelings of stress, subjective feelings of anger, cortisol and SC levels of the participants in the six conditions; n = 30 in each group.

Measure	Stress-sadness	Non-stress-sadness	Stress-reappraisal	Non-stress-reappraisal	Stress-neutral	Non-stress-neutral
Stress (baseline)	3.63 (0.11)^a^	3.67 (0.14)^a^	3.63 (0.11)^a^	3.60 (0.14)^a^	3.63 (0.14)^a^	3.77 (0.13)^a^
Stress (after SI/NSI)	2.73 (0.16)^b^	3.73 (0.14)^a^	2.63 (0.12)^b^	3.83 (0.10)^a^	2.87 (0.16)^b^	3.90 (0.13)^a^
Anger (baseline)	3.93 (0.53)^a^	3.80 (0.46)^a^	3.90 (0.43)^a^	3.77 (0.49)^a^	4.03 (0.53)^a^	3.97 (0.51)^a^
Anger (after AI)	10.80 (0.90)^a^	10.20 (0.99)^a^	10.90 (0.91)^a^	10.30 (0.86)^a^	11.00 (0.79)^a^	9.87 (0.91)^a^
Anger (after AR)	6.70 (0.67)^a^	6.67 (0.74)^a^	6.03 (0.76)^a^	3.73 (0.45) ^b^	6.80 (0.77)^a^	7.10 (0.75)^a^
Cortisol (baseline)	4.98 (0.53)^a^	4.80 (0.57)^a^	5.00 (0.76)^a^	4.94 (0.64)^a^	4.96 (0.59)^a^	4.83 (0.44)^a^
Cortisol (after SI/NSI)	7.65 (0.99)^a^	4.69 (0.81)^b^	7.72 (1.27)^a^	4.97 (0.65)^b^	7.74 (0.94)^a^	4.33 (0.40)^b^
Cortisol (after AR)	5.17 (0.51)^b^	4.44 (0.87)^b^	9.86 (1.92)^a^	4.23 (0.62)^b^	6.06 (0.65)^b^	4.36 (0.47) ^b^
SC (baseline)	13.73 (0.85)^a^	13.16 (0.63)^a^	13.20 (1.03)^a^	13.73 (1.34)^a^	13.56 (1.00)^a^	13.13 (1.11)^a^
SC (after AR)	12.44 (1.22)^b^	11.92 (1.19)^b^	16.52 (1.35)^a^	17.48 (1.67)^a^	17.20 (1.55)^a^	17.37 (1.71)^a^

*Standard errors are in parentheses. Superscripts refer to within-row comparisons. Means with the same superscript are not significantly different at the 0.05 level. SI, stress induction; NSI, non-stress induction; AI, anger induction; AR, anger regulation (sadness induction/cognitive reappraisal).*

### Subjective Feeling of Anger

The 3 (time: at baseline, after anger induction, and after anger regulation) × 2 (stress state: stress induction or non-stress induction) × 3 (regulation strategy: sadness induction, reappraisal induction, or neutral mood induction) repeated-measures ANOVA on self-reported stress showed a significant main effect of time [*F*_(2,173)_ = 136.414, *p* < 0.001, η^2^ = 0.612], but the main effects of stress state and regulation strategy were not significant [*F*_(1,174)_ = 1.570, *p* > 0.05, η^2^ = 0.009; *F*_(2,174)_ = 1.050, *p* > 0.05, η^2^ = 0.012, respectively]. The interaction among time, stress state and regulation strategy was marginally significant [*F*_(2,174)_ = 2.802, *p* = 0.063, η^2^ = 0.031].

Regarding to the marginally significant interaction among time, stress state and regulation strategy, a simple effect analysis (adjusted using the Šidák correction) focusing on the time differences revealed that the subjective anger feelings after anger induction were significantly higher than those at baseline for all groups (*p*s < 0.001), indicating that the anger induction procedure was efficient. In the non-stress condition, the subjective anger feeling after sadness and neutral mood inductions were both significantly higher than those at baseline (*p*s < 0.001), but no significant differences were found between the subjective feeling of anger after reappraisal induction and that at baseline (*p*s > 0.05). In the stress condition, the subjective anger feelings after anger regulation were significantly higher than those at baseline for all stress groups (*p*s < 0.001; see **Figure 3B** and **Table 1**), suggesting that cognitive reappraisal availably decreases subjective anger feelings under non-stress conditions but has no such decreasing effect under stress.

**FIGURE 4:**
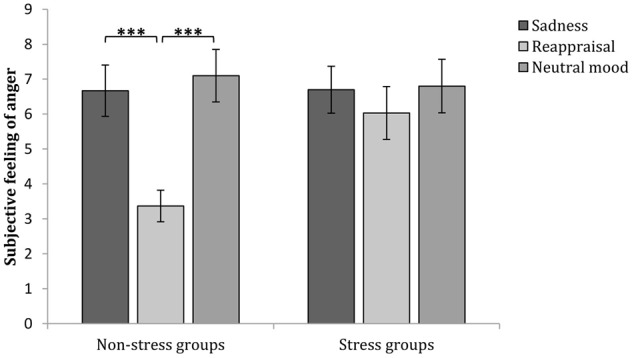
Comparisons of angry feelings after different regulation strategies in the stress and non-stress conditions. The error bars (capped vertical bars) represent ±1 SE. ^∗∗∗^*p* < 0.001.

In addition, a simple effect analysis (adjusted using the Šidák correction) focusing on the regulation strategy differences revealed that no significant regulation strategy differences in subjective anger feeling occurred at baseline or after anger induction between any two groups in the non-stress condition (*p*s > 0.05). However, subjective anger feelings after reappraisal induction were significantly lower than those after sadness induction (*p* < 0.05) and neutral mood induction (*p* < 0.01). In the stress condition, no significant regulation strategy differences were observed at baseline, after anger induction or anger regulation between any two groups (*p*s > 0.05; see **Figures 3B, 4** and **Table 1**). These results indicate two points: First, the effects of anger induction showed no significant group differences; second, stress induction seemed to impair the regulating efficiency of cognitive reappraisal.

### Aggressive Behavior

**FIGURE 5:**
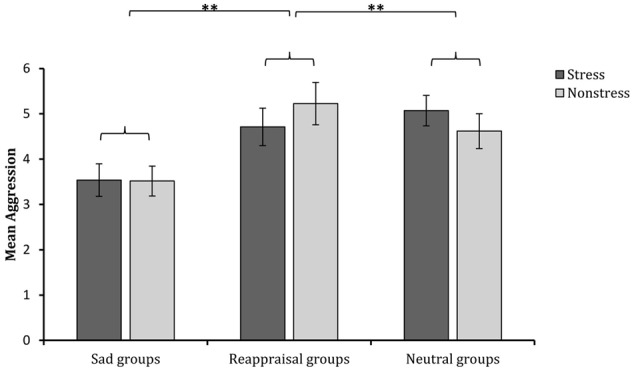
Comparisons of aggressive behavior levels after different regulation strategies in the stress and non-stress conditions. The error bars (capped vertical bars) represent ±1 SE. ^∗∗^*p* < 0.01.

The 2 (stress state: stress induction or non-stress induction) × 3 (regulation strategy: sadness induction, reappraisal induction, or neutral mood induction) univariate ANOVA on aggressive behavior showed that the main effect of stress state was not significant [*F*_(1,174)_ < 1, *p* > 0.05, η^2^ = 0.00001], and the main effect of regulation strategy was significant [*F*_(2,174)_ = 8.595, *p* < 0.001, η^2^ = 0.090). The interaction between stress state and regulation strategy was not significant [*F*_(2,174)_ < 1, *p* > 0.05, η^2^ = 0.009]. Regarding the significant main effect of regulation strategy, multiple comparisons (adjusted using the Šidák correction) showed that the aggressive behavior levels after sadness induction were significantly lower than those after both reappraisal induction (*p* < 0.001) and neutral mood induction (*p* < 0.01; see **Figure 5**). These results indicate that sadness induction is more effective at reducing aggressive behavior than reappraisal and neutral mood inductions, regardless of the stress condition.

### Skin Conductance

The 2 (time: at baseline and after anger regulation) × 2 (stress state: stress induction or non-stress induction) × 3 (regulation strategy: sadness induction, reappraisal induction, or neutral mood induction) repeated-measures ANOVA on SC found that the main effects of time and regulation strategy were both significant [*F*_(1,174)_ = 12.157, *p* < 0.01, η^2^ = 0.065; *F*_(2,174)_ = 3.812, *p* < 0.05, η^2^ = 0.042, respectively]. The interaction among time, stress state and regulation strategy as well as the interaction between time and stress state were not significant [*F*_(2,174)_ < 1, *p* > 0.05, η^2^ = 0.0002; *F*_(1,174)_ < 1, *p* > 0.05, η^2^ = 0.001, respectively]. The interaction between time and regulation strategy was significant [*F*_(2,174)_ = 7.939, *p* < 0.01, η^2^ = 0.084].

**FIGURE 6:**
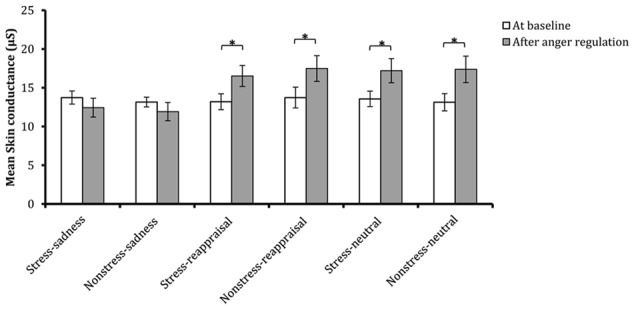
Comparisons of SC at baseline and after anger regulation in each condition. The error bars (capped vertical bars) represent ±1 SE. ^∗^*p* < 0.05.

**FIGURE 7:**
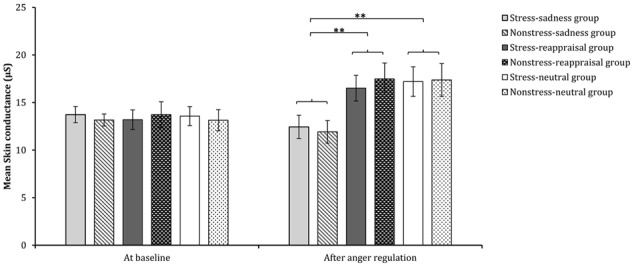
Comparisons of SC across each condition at baseline and after anger regulation. The error bars (capped vertical bars) represent ±1 SE. ^∗∗^*p* < 0.01.

Regarding the significant interaction between time and regulation, a simple effect analysis (adjusted using the Šidák correction) focusing on the time differences showed that the SC levels after reappraisal induction and neutral mood induction were significantly higher than those at baseline (*p*s < 0.01); however, no significant differences were found between the SC level after sadness induction and that at baseline (*p* > 0.05), which indicates that the sadness induction helps the SC level returned to its baseline level (see **Figures 3C, 6** and **Table 1**). In addition, a simple effect analysis (adjusted using the Šidák correction) focusing on the regulation strategy differences showed that the SC level had no regulation strategy differences between any two groups at baseline (*p*s > 0.05), whereas the SC level after sadness induction was significantly lower than those after reappraisal induction and neutral mood induction (*p*s < 0.01; see **Figure 7** and **Table 1**). These results indicate that the sadness induction was more effective at decreasing the SC level than reappraisal and neutral mood inductions, regardless of the stress condition. Furthermore, the SC level after anger regulation was significantly and positively correlated with the level of aggressive behavior (*r* = 0.376, *p* < 0.001).

### Neuroendocrine Results

The 3 (time: at baseline, after stress induction, and after anger regulation) × 2 (stress state: stress induction or non-stress induction) × 3 (regulation strategy: sadness induction, reappraisal induction, or neutral mood induction) repeated-measures ANOVA on cortisol level showed that the main effect of time was significant [*F*_(2,174)_ = 7.652, *p* < 0.01, η^2^ = 0.042], and the main effect of stress state was significant [*F*_(1,174)_ = 11.726, *p* < 0.01, η^2^ = 0.063], but the main effect of regulation strategy was not significant [*F*_(2,174)_ < 1, *p* > 0.05, η^2^ = 0.010]. The interaction among time, stress state and regulation strategy was significant [*F*_(2,174)_ = 3.929, *p* < 0.01, η^2^ = 0.043].

**FIGURE 8:**
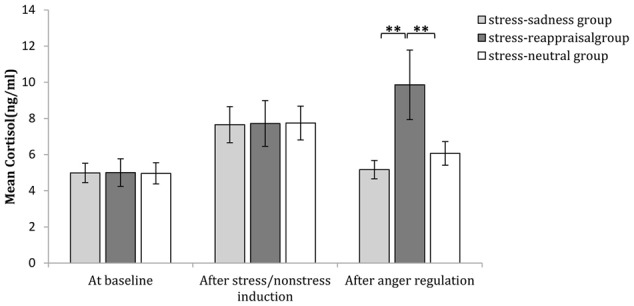
Comparisons of cortisol level regulation strategies after different regulation strategies at baseline, after stress/non-stress induction and after anger regulation in the stress condition. The error bars (capped vertical bars) represent ±1 SE. ^∗∗^*p* < 0.01.

Regarding the significant interaction among time, stress state and regulation strategy, a simple effect analysis (adjusted using the Šidák correction) focusing on the time differences showed that no differences were found in the non-stress condition for all groups (*p*s > 0.05); in stress condition, however, the cortisol levels after stress induction were significantly higher than those at baseline for all groups (*p*s < 0.01), which indicates that the stress manipulation successfully increased cortisol levels. Moreover, neither the cortisol level after sadness induction nor the neutral mood induction significantly differed from baseline (*p*s > 0.05); however, cortisol levels after reappraisal induction were significantly higher than those at baseline (*p* < 0.001; see **Figure 3D** and **Table 1**). In addition, a simple effect analysis (adjusted using the Šidák correction) focusing on the regulation strategy differences showed that in non-stress condition, the cortisol level showed no differences at any of the three time points (*p*s > 0.05); in the stress condition, the cortisol level did not differ at baseline or after stress induction between any two groups (*p*s > 0.05); however, the cortisol levels after reappraisal were significantly higher than those after sadness induction (*p* < 0.01) and neutral mood induction (*p* < 0.05; see **Figures 3D, 8** and **Table 1**). These results indicated that the effects of stress induction did not significantly differ by group, and the reappraisal induction maintained the cortisol level relative to sadness induction and neutral mood induction. Moreover, we found a significantly negative relationship between cortisol concentration and the self-reported stress score after stress/non-stress induction (*r* = -0.150, *p* < 0.05).

In addition, we conducted a similar analysis on the sAA levels but did not find any main effects [time: *F*_(2,173)_ = 1.951, *p* > 0.05, η^2^ = 0.022; stress state: *F*_(1,174)_ < 1, *p* > 0.05, η^2^ = 0.003] or interaction effects [time × stress state: *F*_(2,174)_ < 1, *p* > 0.05, η^2^ = 0.005; time × regulation strategy: *F*_(4,174)_ < 1, *p* > 0.05, η^2^ = 0.008; time × stress state × regulation strategy: *F*_(4,174)_ < 1, *p* > 0.05, η^2^ = 0.006], perhaps because the timing of our assessment was not optimal for the relatively rapid response of sAA (Maruyama et al., 2012).

## Discussion

The present study compared the effects of two strategies for regulating anger and its related aggressive behavior under stress or non-stress conditions: reappraisal based on cognitive ER theory and the SCA concept based on the emotion theory of mutual promotion and mutual restraint in traditional Chinese medicine. The findings can be summarized into two key points: First, cognitive reappraisal effectively decreased subjective feelings of anger under non-stress conditions, but this effect disappeared under acute stress; second, the SCA strategy reduced individual aggression (primarily characterized by the intensity level set by the participants to punish others) after they were provoked, regardless of the stress condition. This “all-day” efficiency was also reflected in participant SC (i.e., the SCA strategy also relatively decreased individual SC after being irritated under both stress conditions). Generally, these results provide evidence supporting our main hypothesis that the efficiency of the SCA regulation strategy is less influenced by stress relative to cognitive reappraisal. Furthermore, three points should be noted. First, the stress manipulation in the present study involved only acute physiological stress. Thus, our findings should not be arbitrarily generalized to conditions of chronic stress. Second, the present study investigated only the regulation efficiency of state sadness on state anger or aggressive behavior. Therefore, the conclusions should not be simply extended to traits of depression or aggression. Third, although state sadness decreased anger-related aggressive behaviors, issues concerning the regulation effects of long-term sadness induction and whether long-term sadness has adverse effects require further examination.

The findings of the present research are consistent with previous research. First, a previous study found that cognitive reappraisal fails to regulate fear conditioning under stress (Raio et al., 2013). Furthermore, regarding the effects of the SCA strategy, our previous research showed that inducing sadness among participants (relative to fear and neutral emotions) after they were provoked decreased their aggression (Zhan et al., 2015). The present study successfully replicated previous studies and directly compared cognitive reappraisal with the SCA strategy after controlling for the object of regulation (i.e., anger and aggressive behavior) and the means of regulation (i.e., both cognitive reappraisal and the regulatory sadness emotion were induced by watching videos) across conditions. This approach demonstrated the superiority of the SCA strategy with regard to resisting stress interference.

The differences between cognitive reappraisal and the SCA strategy in resisting stress might be related to the hormonal changes driven by the stress responses of HPA axis arousal, which might further impair the functions of the PFC. This study used the CPT to manipulate stress and effectively increase individual cortisol levels in keeping with numerous previous studies in psychoneuroimmunology, neurodevelopment, PTSD and animal models that have revealed the destructive effects of cortisol on PFC function (Hartley and Adams, 1974; Carrion et al., 2010; Wolf, 2016; Goldfarb et al., 2017). Cortisol promotion via stress likely rapidly disrupts PFC network connections and markedly impairs PFC function during ER. The frontal regions that regulate one’s thoughts, actions and emotions might be particularly sensitive to the effects of cortisol. Cortisol regulates its own release via a negative feedback loop in the central nervous system (CNS), where it binds to the glucocorticoid receptor (GR) distributed widely in the PFC (Fuster, 2008). Furthermore, many studies have demonstrated that stress rapidly disrupts PFC network connections and markedly impairs PFC function, including the performance of tasks that require complex and flexible thinking (Hartley and Adams, 1974; Wolf, 2016; Goldfarb et al., 2017). Studies of youth with posttraumatic stress symptoms (PTSSs) have found a significant negative association between pre-bedtime cortisol levels and left ventral PFC gray volumes, suggesting that these youth have more detrimental exposure to cortisol and therefore more negative PFC development effects (Carrion et al., 2010). Moreover, a deleterious effect of cortisol was found with regard to the retrieval of previously learned material, with specific impairments to working memory (Lupien et al., 1999, 2007; Young et al., 1999; Cahill et al., 2003). Animal research has also indicated that chronic behavioral stress can lead to dendritic reorganization in the medial PFC (Wellman, 2001; Radley et al., 2004) and that exposure to stress-induced cortisol elevation can impair prefrontal behavioral control in monkeys (Lyons et al., 2000). With regard to the neural correlates of cognitive ER, the PFC is a crucial brain area with regard to regulation success (Kohn et al., 2014; Etkin et al., 2015). Based on neuroimaging studies, the ventrolateral PFC (VLPFC) plays a major role in generating and appraising emotion and affect (Ochsner and Gross, 2005; Phillips et al., 2008; Kohn et al., 2014; Etkin et al., 2015). In contrast, the dorsolateral PFC (DLPFC) is functionally characterized by engaging in purely cognitive tasks such as working memory and selective attention. This area is strongly associated with the cognitive control of emotion processing, regulating emotion generated by the amygdala, insula and VLPFC (Barbey et al., 2013; Kohn et al., 2014; Etkin et al., 2015). Because the HPA axis activated by stress might impair the cognitive functions of the PFC and further undermine cognitive regulation (Raio et al., 2013), we speculated that the acute stress exposure of the present study would impair individuals’ advanced functioning of the frontal region to regulate anger. In contrast to the top-down strategy of cognitive reappraisal, the SCA strategy involves using one type of emotion to counteract another. First, sadness might be able to counteract anger because of the dissimilarity of these emotions with regard to their cognitive and emotional elements. According to a meta-analysis on the brain activation patterns of different emotional categories, anger preferentially engages cortical processes that support an “external orientation/object-focused” schema that is characterized by goal-driven responses for which objects and events in the world are in the foreground, whereas sadness engages cortical patterns that support an internal orientation/homeostatic-focused schema that is characterized by an orientation toward immediate somatic or visceral experience that prioritizes the processing of interoceptive and homeostatic events (Wager et al., 2015). Specifically, anger is primarily characterized by the strong activity of dorsal attention and the strong co-activation of the visual-to-frontoparietal cortex as well as subcortical co-activation, indicating a strongly goal-driven attentional component with central cerebellar involvement for strong sensorimotor integration. By contrast, sadness cortical patterns are prominently characterized by a profound lack of co-activation between the cortical and subcortical cerebellar/brainstem networks and strong cerebellar-brainstem co-activation, suggesting a weak activation of the integrated planning/action systems (dorsal attention/cerebellar) and strong reflexive cerebellar-brainstem responses that operate without co-activation with the cortex (Wager et al., 2015). Thus, the internally oriented rather than externally oriented neural network activated by sadness induction likely relieves the neural system activated by provocation, thereby reducing aggression level. Second, limited self-control resource theory might explain why stress did not affect the efficiency of the SCA strategy. This theory suggests that when a self-control resource is over consumed, ego depletion is initiated, which can adversely affect an individual’s emotional, cognitive, and behavioral performance (Muraven et al., 1998; Baumeister and Vohs, 2007; Barlett et al., 2016). Accordingly, because the SCA strategy requires fewer self-control resources than reappraisal, this strategy is not invalidated even under stress conditions that result in ego depletion. Thus, the SCA strategy depends less on the PFC, which is less sensitive to stress hormone responses (e.g., cortisol).

The present study suggests an interesting phenomenon. Although the cortisol levels of the three stress groups were significantly higher than those of the three non-stress groups (which confirmed the efficiency of stress manipulation in this study [i.e., the CPT]) and no significant differences were found among the three stress groups with regard to cortisol levels before watching the video to regulate anger, after watching the video, the cortisol levels of the stress-reappraisal group increased, whereas the those of the stress-sadness group and the stress-neutral group decreased (although the increasing and decreasing trends were not significant when contrasting the cortisol level before and after watching the video within each condition; the cortisol level of the stress-reappraisal group after watching the movie was significantly higher than that of the stress-sad and stress-neutral groups). These differences suggest that cognitive reappraisal under stress might interact with previous stress states. Although the reappraisal effort aroused by watching a video of cognitive reappraisal could not form a new stressor, it was at least able to maintain the physiological stress relative to sadness and neutral emotion induction. This finding indicates that cognitive reappraisal under stress is not only a matter of cognitive resource depletion but also might interact with the stress response and maintain or even exacerbate the stress influence. Compared with watching the cognitive reappraisal video, watching the sad and neutral videos under stress did not cause individual cortisol levels to increase or cause them to remain high, indicating that the interacting intensity between the sadness induction and neutral emotion induction conditions and stress induction were both less than cognitive reappraisal. The cortisol levels of an individual under stress after watching a sad video or a neutral video did not significantly differ, implying that cortisol returned to baseline after mood induction. However, aggression level was significantly lower after watching the sad video than after watching the neutral video, which excludes the possibility that the SCA effect functions by imposing on cortisol responses.

Another difference between cognitive reappraisal and the SCA strategy is related to the target of regulation. Cognitive reappraisal under the non-stress condition effectively decreased subjective feelings of anger but did not reduce aggression or related physical arousal (SC), whereas the SCA strategy alleviated aggression and related physical arousal (SC) but did not relieve subjective feelings of anger. Anger is often but not always associated with aggression or relevant physiological indices. Some studies have shown that self-reported anger is weakly correlated with aggression in both laboratory (e.g., Kassinove et al., 2002; Giumetti and Markey, 2007) and real-life situations (Nesbit et al., 2007). However, the relationship between self-reported anger and aggression or the cognitive-physiological discordance of anger might be influenced by several variables that have been largely unexplored by empirical research (Hortensius et al., 2012). One possible explanation for these discrepancies in the present study might be the cognitive or distractive characteristic of the reappraisal or SCA regulation strategies. Specifically, the induction of cognitive reappraisal functioned by changing the participants’ cognitive attitudes toward the anger event (being insulted by another participant), which might be closely related to self-reported anger but relatively unrelated to the physical arousal and aggressive behavior measured via SC and the aggressive test paradigm. In contrast, the SCA strategy might function as distraction and counteraction approaches. The induction of sadness did not change participants’ attitudes toward the angry event that they encountered, but it might have interrupted and counteracted the mindset that an angry individual typically maintains. Nevertheless, we cannot arbitrarily claim that sadness reduces only aggression but not subjective feelings of anger. In fact, the subjective feeling of anger consists of a cognitive component and an emotional experience component. The former might be characterized more easily by self-reported anger, whereas the latter concerns the aggressive impulses in the present study. Because aggressive behavior was assessed in a situation with specific anger cues (TAP) and directly driven by previous provocation, this aggression might be more of an emotional experience related to anger. Therefore, decreasing aggression after sadness induction implies the potential function of sadness in regulating the angry experience component of the subjective anger feeling. However, the regulation effects of sadness induction on the subjective feeling of anger or self-reported anger require further confirmation.

## Limitations

Several limitations of this study should be considered. First, in this study, all participants were randomly selected from university students, this may limit the generalizability of our results to children, old people and especially populations that are without academic degree. However, given the movie materials that the SCA strategy used are so easy to be understood that even the individuals without much education have no any difficulty to get the meaning and experience the corresponding emotion, we think the influence of academic degree could be minor if there were any, or at least, the influence of academic degree in our strategy should be much more less than the cognitive reappraisal strategy that require relatively complicated cognitive skill to change one’s mental representation way for the unfavorable situations. Second, we primarily focused on temporal state emotion and behavior. Trait personality and characteristics (e.g., trait aggression, chronic stress, and depression) should be taken into account in future studies. Third, our results did not indicate a significant relationship between the sAA and anger-related response. However, we observed a marginally significant positive correlation between sAA and cortisol. Both noradrenergic and glucocorticoid responses to stress and their interacting influence in the brain might impair the efficiency of reappraisal in decreasing feelings of anger. Specifically, glucocorticoids (e.g., cortisol) exaggerate catecholamine (e.g., noradrenaline as measured by sAA) actions in the PFC by blocking the extraneuronal catecholamine transporters on the glia that clear the extrasynaptic space of catecholamine (Arnsten, 2009). Studies of the neural mechanisms that underlie stress-induced cognitive regulation impairment should continue to address this issue. Finally, the supporting evidence for the SCA strategy remains at the behavioral level with speculation about neuroendocrine responses. Precise neural correlates of the interaction between sadness and anger should be addressed in future research.

## Conclusion

The present study found that cognitive reappraisal decreased individuals’ self-reported anger and sadness induction reduced aggressive behavior and SC under non-stress conditions. Under acute stress conditions, cognitive reappraisal did not successfully relieve angry feelings and was associated with higher cortisol levels (relative to the non-stress condition), whereas sadness induction showed the same effect as that in the non-stress condition. These results replicate previous findings regarding the failure of cognitive ER under stress (Raio et al., 2013), suggesting that the cortisol response driven by the HPA axis arousal destroys PFC function and further impairs the efficiency of cognitive regulation. Above all, our findings provide an important step in the investigation of a neuroendocrine convergent extension for the efficiency of the SCA strategy that is less reliant on PFC function, implying an approach that requires relatively less cognitive control for ER.

## Ethics Statement

This study was approved by the Institutional Review Board at Capital Normal University. In compliance with the principles of the Declaration of Helsinki, all participants provided written informed consent prior to the start of experimentation.

## Author Contributions

JZ performed experimental design, implement of experiment, analysis on all samples, interpreted data, wrote manuscript and acted as first author; JL had substantial contributions to the conception or design of the work, supervised experimental design, interpreted data, edited manuscript and acted as the first corresponding author; CL supervised development of work, helped in sample collection, data interpretation, manuscript evaluation and acted as second corresponding author; JR supervised development of work, helped in experiment design, data interpretation and manuscript evaluation and acted as third corresponding author; XFW participated in data analysis, data interpretation and manuscript evaluation; JF supervised experimental design, helped to analysis all samples and interpreted data, evaluated and edit the manuscript; JYG participated in experimental design, manipulated test of the biochemical indicators, helped in the analysis of data and edited manuscript; JSZ supervised development of work, participated in experimental design and data interpretation, helped to evaluate and edit the manuscript.

## Conflict of Interest Statement

The authors declare that the research was conducted in the absence of any commercial or financial relationships that could be construed as a potential conflict of interest.

## Supplementary Material

We also used all three cortisol measures to calculated the area under the curve with respect to ground (AUCg; see Pruessner et al., 2003, for the formula), which was a cumulative index of total cortisol output in response to various laboratory challenges, the corresponding figure was presented as Supplementary Materials.

FIGURE S1 Comparisons of AUCg levels of cortisol after different regulation strategies in the stress and non-stress conditions. The error bars (capped vertical bars) represent (-1)/(+1)SE. ^∗∗^*p* < 0.01.
