# Supplementary figures and images for: Regulating Anger under Stress via Cognitive Reappraisal and Sadness
Source: Front Psychol. 2017 Aug 14;8:1372. doi: 10.3389/fpsyg.2017.01372 (PMC5557741; doi:10.3389/fpsyg.2017.01372)

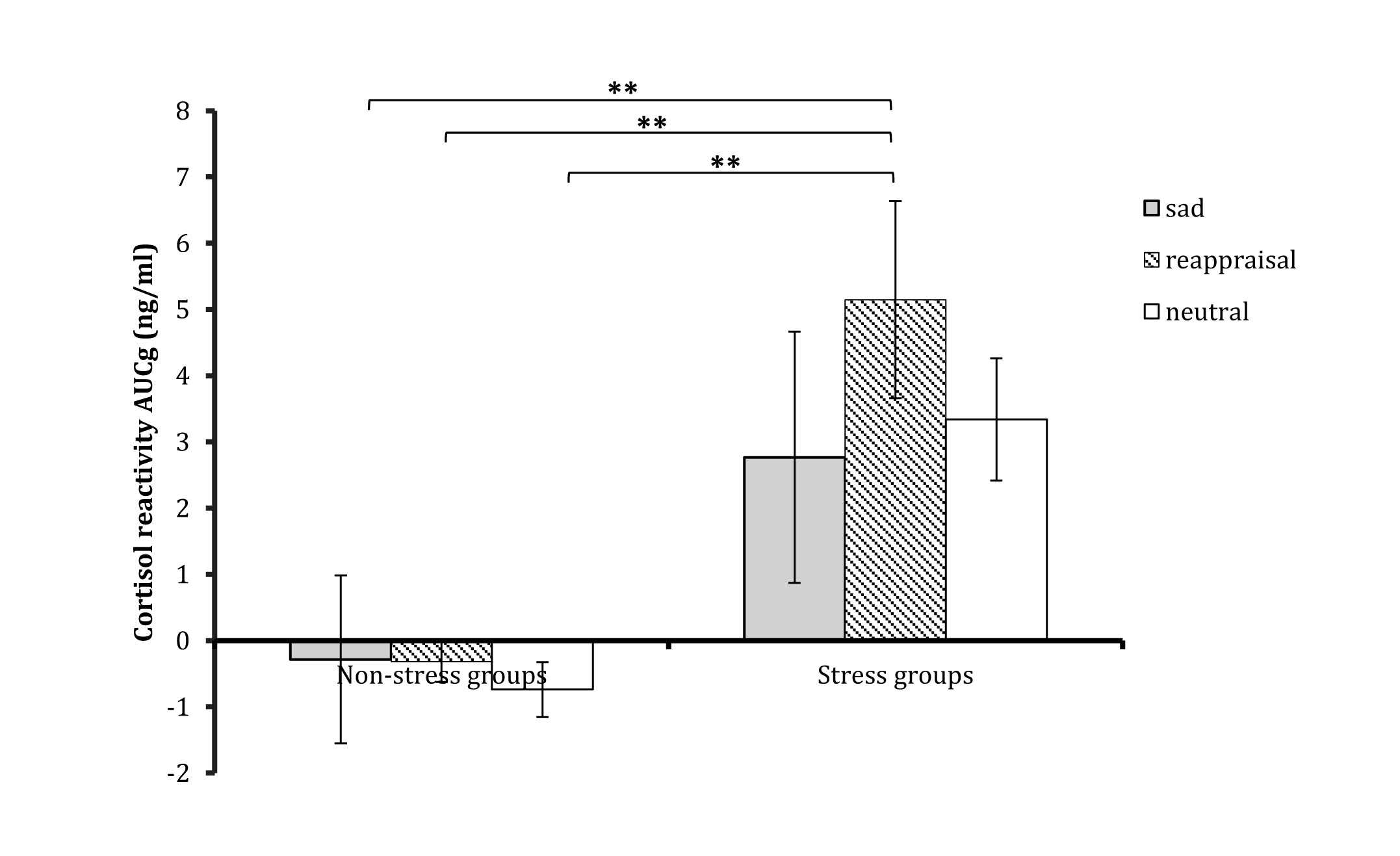

Supplement: FIGURE S1 — Comparisons of AUCg levels of cortisol after different regulation strategies in the stress and non-stress conditions. The error bars (capped vertical bars) represent (-1)/(+1)SE. ∗∗p < 0.01. [file Image_1.JPEG]
